# An emerging role of the cellular prion protein as a modulator of a morphogenetic program underlying epithelial-to-mesenchymal transition

**DOI:** 10.3389/fcell.2014.00053

**Published:** 2014-09-18

**Authors:** Mohadeseh Mehrabian, Sepehr Ehsani, Gerold Schmitt-Ulms

**Affiliations:** ^1^Tanz Centre for Research in Neurodegenerative Diseases, University of TorontoToronto, ON, Canada; ^2^Department of Laboratory Medicine and Pathobiology, University of TorontoToronto, ON, Canada

**Keywords:** epithelial-to-mesenchymal transition, function, phenotype, prion protein, ZIP transporter

## Abstract

Knowledge of phenotypic changes the cellular prion protein (PrP^C^) contributes to may provide novel avenues for understanding its function. Here we consider data from functional knockout/down studies and protein–protein interaction analyses from the perspective of PrP's relationship to its ancestral ZIP metal ion transporting proteins. When approached in this manner, a role of PrP^C^ as a modulator of a complex morphogenetic program that underlies epithelial-to-mesenchymal transition (EMT) emerges. To execute EMT, cells have to master the challenge to shift from cell-cell to cell-substrate modes of adherence. During this process, cell-cell junctions stabilized by E-cadherins are replaced by focal adhesions that mediate cell-substrate contacts. A similar reprogramming occurs during distinct organogenesis events that have been shown to rely on ZIP transporters. A model is presented that sees ZIP transporters, and possibly also PrP^C^, affect this balance of adherence modes at both the transcriptional and post-translational levels.

## Introduction

To someone new to the prion literature, it might be confusing that on the one hand some researchers lament the absence of a known function of the cellular prion protein (PrP^C^), while on the other hand, there seems to be a myriad of roles assigned to this protein (Steele et al., 2007; Aguzzi et al., 2008; Zomosa-Signoret et al., 2008). Indeed, over the past few decades, mouse models harboring a deletion of the prion gene have revealed some interesting and highly diverse phenotypes, including (i) defects in the circadian rhythm (Tobler et al., 1996), (ii) abnormal development of infrapyramidal mossy fibers (Colling et al., 1997), (iii) a reduced capacity to generate hematopoietic stem cells (Zhang et al., 2006), and (iv) a peripheral neuropathy that is most conspicuously characterized by a myelin maintenance defect (Bremer et al., 2010). Due to the complex nature of the biology underlying any of these phenotypic changes, none of them immediately suggest how PrP^C^ might be involved. It also does not help that each of them requires a unique environment not easily accessible by experimental means. The only phenotype for which a wealth of available data has provided a near-complete molecular explanation constitutes the well-known resistance of PrP-knockout mice to infection with prion disease (Sailer et al., 1994).

This mini-review will add to this body of literature by shining a spotlight on the possibility that the prion protein may, in some cellular contexts, operate in signaling events that influence a shift from cell-cell to cell-substrate modes of adherence and play, for example, a critical role during EMT. In light of the emphasis of this special issue on research that explores the function of the prion protein, a brief clarification of terms and concepts may be helpful.

## The physiological role of a protein—phenotypic change vs. function

Although intuitively appealing, applying the concept of “function” to a protein can be a difficult undertaking. Any protein that emerges from ribosome-mediated translation enters into a unique molecular life-cycle that ultimately comes to an end when the protein is degraded. During this cycle, a protein encounters many other molecules that may impact its own biology. Some of these may, for instance, facilitate its post-translational maturation and folding or influence its sorting and trafficking. The consequences of molecular interactions may be equidirectional, orthogonal or opposite with regard to a specific outcome. They may also possess other characteristics that complicate their study; for example, they can be transient or long-lasting, and can qualitatively or quantitatively change over time. The net effect of these molecular interactions will not only depend on the physicochemical properties of the protein of interest itself, but also on the status of the cell or multicellular entity into which it was born. The latter will, in turn, be influenced by the characteristics and locations of all other molecules present at a given time. For example, there are several proteins (e.g., WNT5A, RACK1, SIRT1 and several F-box proteins) whose expression levels appear to correlate with the risk to acquire certain cancers, yet the same proteins have been observed to confer protection against cancers in other paradigms (McDonald and Silver, 2009; Lin and Fang, 2013; Li and Xie, 2014; Wang et al., 2014).

A phenotypic change caused by a protein can be discerned if its molecular interactions cumulatively change the biology of the experimental paradigm in a way that is detectable (Crusio, 2002). It follows that the description of such a phenotypic change only becomes meaningful if a precise account of the experimental paradigm is also provided.

What is the relationship between phenotypic change and function? The term “function” seems overused in the protein literature in general and the prion literature in particular. Here, the use of this term will be restricted to an immediate and context-independent role of a protein (e.g., the capacity of substrate phosphorylation by a kinase) and will not be employed to capture the contribution of a protein to a broad and indirect phenotypic change (e.g., mitosis).

When viewed in this light, the body of literature dealing with functional assignments of the prion protein begins to look considerably less controversial. It comes as no surprise that a protein studied as extensively as PrP^C^ has been observed to contribute to several biological processes that give rise to diverse phenotypes. While the phenotypic changes a protein causes are not to be confused with its function, once the function is known, it will hopefully reconcile a majority of these phenotypic changes with a consistent molecular and mechanistic explanation.

## EMT, yet another phenotypic change the prion protein may contribute to—but not its function

**Figure 1:**
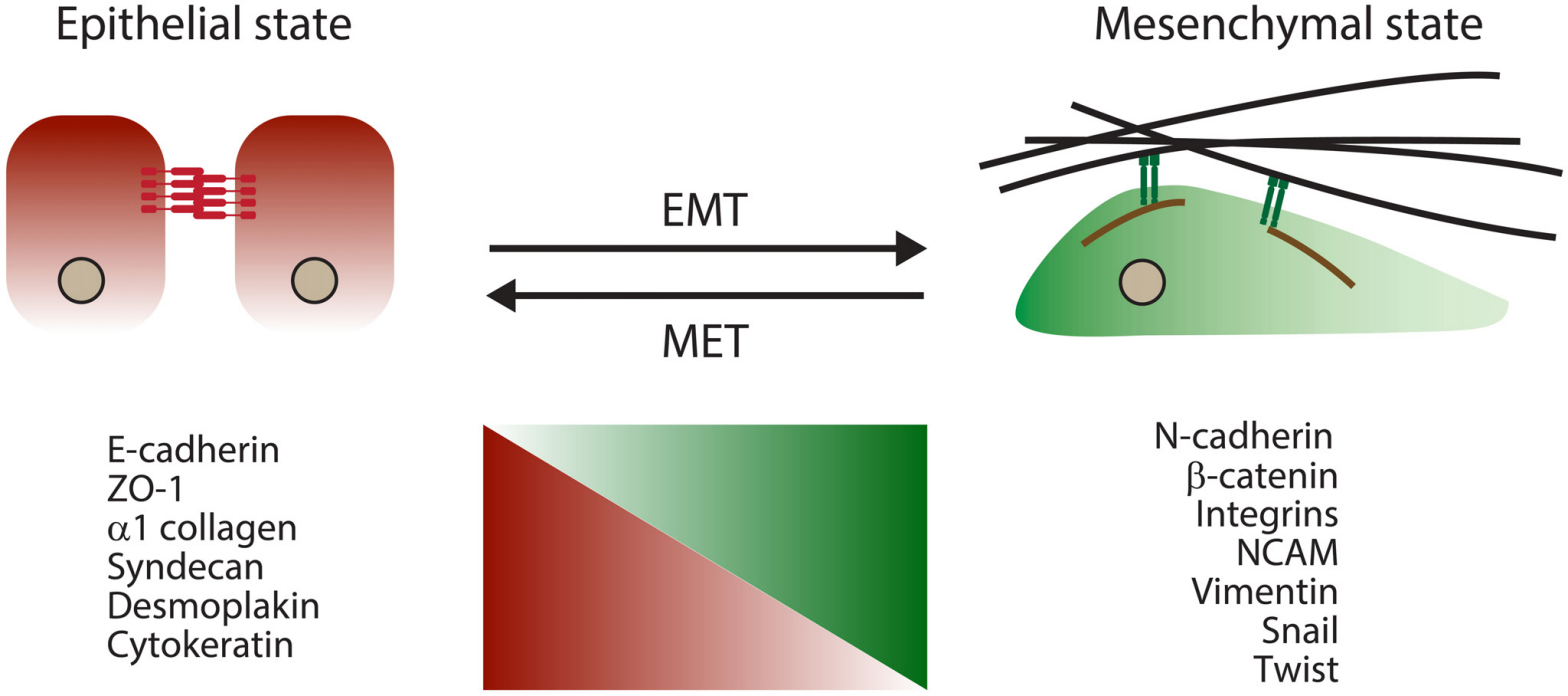
**Schematic outlining key morphological and molecular changes that accompany EMT**.

The term EMT refers to a complex morphogenetic reprogramming of cells observed during their transition from epithelial to mesenchymal phenotypes. EMT is naturally initiated in cells during distinct stages of development and wound healing (Lim and Thiery, 2012). Similar morphogenetic changes also take place during organogenesis and in pathological conditions, including fibrosis and cancers, where their occurrence most often correlates with a higher metastasis propensity (Thiery et al., 2009). During the EMT process, strong epithelial cell-to-cell contacts in the form of adherens junctions gradually give way to more transient cell-to-matrix connections evident in cultured cells (referred to as focal adhesions). As adherens junctions are dissolved, functional levels of E-cadherin, their main molecular constituent, are also reduced. Consequently, cells acquire a more fibroblast-like morphology and gradually rely on a more dynamic molecular network involving integrins for cell–matrix interactions (Lamouille et al., 2014). During the cellular reprogramming that accompanies EMT, the levels and/or activities of many molecules change (Figure 1).

The notion of an involvement of PrP^C^ in the morphogenetic program underlying EMT emerged recently from several independent strands of investigation, including (1) observations in cancer paradigms that predominantly see a role of PrP^C^ as a promoter of cellular invasiveness and malignancy; (2) morpholino-based manipulations of PrP levels undertaken in zebrafish; (3) a subset of available PrP protein–protein interaction data; and (4) commonalities between PrP and the subbranch of ZIP transporters from which it descended. Finally, pilot data generated in mammalian cells in which the expression of PrP^C^ was eliminated using CRISPR/Cas gene editing tools (Hsu et al., 2014) are also consistent with a role of PrP^C^ as a modulator of a cellular program that defines the balance between cell-cell and cell-substrate adhesion (unpublished observations, Schmitt-Ulms laboratory). Therefore, when asked to contribute to this special issue, we decided to take the opportunity to further investigate the merits of an involvement of PrP^C^ in EMT based on published reports. Needless to say, no claim is made that an involvement of PrP^C^ in EMT, even if further substantiated experimentally, would represent its physiological function. Instead, it is hoped that this contribution might further stimulate research that may uncover the function of PrP^C^.

### Evidence for a role of PrP^C^ in EMT in the cancer literature

In light of the central role that EMT plays in the cellular biology that governs tumor malignancy, it may not be surprising that some support for a possible role of PrP^C^ in EMT can be found in the cancer literature. Several groups have reported that the prion protein is upregulated in a subset of human cancers ranging from colorectal and gastric cancers to breast cancer and glioblastomas (Mehrpour and Codogno, 2010). Furthermore, a positive correlation has been reported between the upregulation of PrP^C^ and the tumorigenicity of some cancer cells, suggestive of a more causative involvement. For instance, in one of the first reports that tied PrP^C^ to cancer biology, the overexpression of PrP^C^ was observed to promote invasive and metastatic properties of gastric cancer cell lines (Pan et al., 2006), and a more recent report suggested that high PrP^C^ levels can be predictive of disease recurrence in colorectal cancers (Antonacopoulou et al., 2010). Overall, a broad consensus seems to be emerging that PrP^C^ can promote key indicators of malignancy in several cancer paradigms. However, less information and agreement exist at this time on the signaling pathways and molecular events involved. Several observations suggest that PrP^C^ may modulate signaling downstream of TGFβ. For example, it was reported that PrP^C^ promotes the release of certain matrix metalloproteinases (Pan et al., 2006; Wurm and Wechselberger, 2006) that are activated during TGFβ signaling and are known to promote the breakdown of the extracellular matrix, thereby leading to cell detachment. Also, researchers have proposed that the serine/threonine kinases Erk1/2 may play a critical role in the cancer-related signaling cascade emanating from PrP^C^. This conclusion was drawn on the basis of data documenting that small-molecule inhibitors against key components of the mitogen-activated protein (MAP) kinase signaling pathway were able to rescue invasive and metastatic characteristics contributed by PrP^C^(Pan et al., 2006; Du et al., 2013). Although an inverse relationship between E-cadherin and PrP^C^ levels has been observed (Du et al., 2013), whether this relationship is merely correlative or if PrP^C^ causally influences E-cadherin biology has, to our knowledge, not yet been addressed in the aforementioned cancer paradigms.

### Zebrafish PrP knockdown models

To date, the most dramatic phenotypic change associated with PrP deficiency was not reported for the mouse prion protein but for one of its two orthologs in zebrafish (Malaga-Trillo et al., 2009). More specifically, the morpholino-based knockdown of PrP1 (but not PrP2) was observed to cause an arrest of zebrafish embryogenesis at the gastrula stage in the course of cellular rearrangements that rely on EMT. Interestingly, the PrP1-deficient embryos do not appear to suffer from an inability to initiate EMT; rather, they are impaired in their ability to fully execute a cell migration program that is limited to a small number of cells and requires re-establishing cellular contacts following their migration (Malaga-Trillo et al., 2009). Whereas in normal embryos PrP1 was shown to activate Src family kinases (SFKs) and modulate the post-transcriptional E-cadherin biology, both of these activities were impaired in PrP1-deficient embryos. A follow-up investigation revealed that several features within PrP1, including the repeat domain, globular domain, N-glycosylation sites and GPI anchor attachment signal, influence the proper positioning of PrP1 at cell-cell contacts (Solis et al., 2013). Importantly, the phenotype does not appear to reflect a functional specialization or idiosyncrasy of zebrafish PrP1 because it could be rescued by the introduction of mammalian PrP^C^ (Malaga-Trillo and Sempou, 2009; Malaga-Trillo et al., 2009).

### Protein–protein interactions of PrP^C^ with a known EMT connection

Although there are divergent views regarding the significance of protein–protein interactions of PrP^C^ (Watts and Westaway, 2007; Aguzzi et al., 2008; Rubenstein, 2012), there appears to be a broad consensus that PrP^C^ exerts some of its biological role through its affiliation with caveolae and specialized membrane domains referred to as lipid rafts (Naslavsky et al., 1997; Mouillet-Richard et al., 2000). Furthermore, there is agreement that the molecular microenvironment of PrP^C^ in neurons is dominated by cell adhesion molecules that include neural cell adhesion molecule (NCAM) (Walsh et al., 1989), L1, integrins and non-integrin laminin receptors known to modulate cell-to-substrate contacts (Gauczynski et al., 2001; Watts et al., 2009). Finally, there is a broad consensus that signals emanating from PrP^C^ can lead to Fyn activation (Mouillet-Richard et al., 2000; Santuccione et al., 2005; Toni et al., 2006; Pantera et al., 2009; Tomasi, 2010; Um et al., 2012).

Several of these physiological interactors of the prion protein have been implicated in EMT. NCAM, for example, has recently been shown to be a critical regulator of EMT (Lehembre et al., 2008; Evseenko et al., 2010). Specifically, (i) increases in the levels of NCAM and (ii) a redistribution of NCAM that may involve its detachment from fibroblast growth factor (FGF) receptors and recruitment into caveolae and/or raft-like domains (Niethammer et al., 2002; Santuccione et al., 2005), have been recognized as early steps during EMT (Lehembre et al., 2008). Not surprisingly, these changes to the cellular NCAM pool and the concomitant upregulation of functional integrins occurring during EMT lead to increases in signaling through Fyn (Lehembre et al., 2008). Moreover, αV-containing integrins, another family of PrP^C^ interactors (Watts et al., 2009), are critical for activation of the EMT master regulator TGFβ 1 (Munger et al., 1999; Mu et al., 2002). Mice harboring point mutations in the αV integrin “RGD” recognition motif within the TGFβ 1 preprotein (TGFβ1^RGE/RGE^) were shown to phenocopy TGFβ 1-null mice (Yang et al., 2007). As an additional example, β 1 integrin-L1 complexes have long been known to play a role in controlling cellular migration processes. More recently, L1 has been linked to EMT and a molecular biology that determines cancer invasiveness (Kiefel et al., 2012). Thus, the stage, players and activities that PrP^C^ is surrounded by are familiar to the research community studying EMT.

### Function of closest evolutionary relatives of PrP

Members of the mammalian prion protein family (PrP^C^, Dpl and Sho) were recently shown to have evolved from the family of ZIP (Zrt-, Irt-like Protein) metal ion transporters (Schmitt-Ulms et al., 2009), with up to 30% amino acid sequence identity observed in some pair-wise comparisons of PrP and ZIP sequences. Subsequent work established that the founding event of the prion gene family coincided with the speciation of early vertebrates around 500 million years ago. This event relied on a genomic rearrangement that involved the retroinsertion of an ancient ZIP transcript and, probably, was mediated by retrotransposition elements (Ehsani et al., 2011). Amino acid sequence comparisons revealed that PrP is most similar to ZIPs 5, 6 and 10, which constitute a distinct sub-branch within this gene family that consists of 14 paralogs in humans (Schmitt-Ulms et al., 2009). The primary cellular function of ZIP transporters is the import of zinc and other divalent cations into the cytosol (Lichten and Cousins, 2009). The PrP-like (PL) domains within ZIPs 5, 6 and 10 represent their N-terminal ectodomains and resemble PrP with regard to orientation and relative distance to their downstream membrane anchorage sites. The PL domain most likely fulfills a role as a modulator of the cation import channel to which it is attached (Ehsani et al., 2012).

Interestingly, ZIP6 deficiency in zebrafish embryos has been reported to give rise to a gastrulation defect that is similar to the aforementioned defect observed in PrP1-deficient zebrafish (Yamashita et al., 2004). Subsequent work documented that manipulation of ZIP6 expression levels in mammalian cells leads to equidirectional changes in levels of E-cadherin expression (Shen et al., 2009). The close *Drosophila* ZIP ortholog Fear-Of-Intimacy (FOI) (Mathews et al., 2005) has similarly been shown to play a role in EMT-like morphogenetic cell movements underlying both gonad and trachea formation (Godt and Tepass, 2003; Van Doren et al., 2003). More specifically, the zinc transport activity of FOI was shown to be essential for post-transcriptional stabilization of E-cadherin expression, with loss-of-function mutants of FOI leading to strongly reduced levels of functional E-cadherin. This did not preclude initiation of cell migration, but interfered with the coalescence of cells following their migration (Mathews et al., 2006). Additional support for the notion that ZIP proteins may affect EMT-like signaling came from a recent genetic report of a family afflicted with a subtype of myopia, a leading cause of blindness in humans, which identified a nonsense mutation in the ZIP5 gene to co-segregate with the phenotype (Guo et al., 2014). Follow-up biochemical work led the authors to observe that ZIP5 deficiency interfered with signaling downstream of TGFβ.

## Model of PrP/ZIP E-cadherin modulation

**Figure 2:**
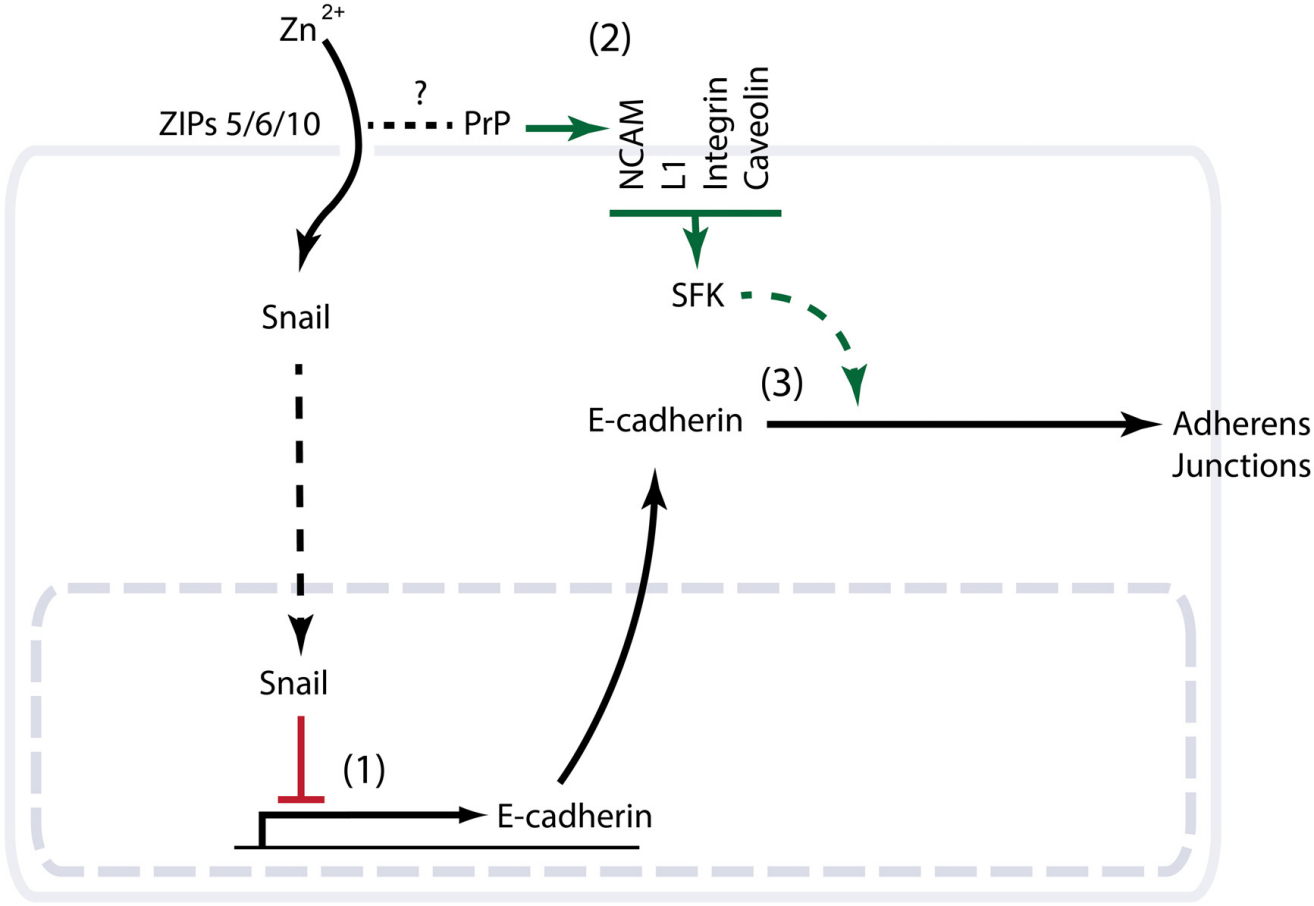
**Schematic summarizing evidence consistent with a role of PrP^C^ in EMT**. (1) The closest molecular cousins of PrP from the ZIP zinc transporter family possess a documented role in EMT. Knockout or knockdown phenotypes of the respective ZIPs in zebrafish, *Drosophila* or mammalian cells demonstrate a critical role of ZIP-dependent zinc import in the transcriptional control of the E-cadherin gene. Note that whereas in the zebrafish EMT paradigm this transcriptional control is orchestrated by the EMT master regulator Snail and suppresses E-cadherin expression, the zinc import function of the *Drosophila* FOI gene was observed to promote E-cadherin transcription during gonad organogenesis (not shown). (2) Multiple interactors of PrP^C^ with a known ability to modulate the activity of Fyn (a Src family kinase) are independently known to play a role in EMT. (3) PrP knockdown in zebrafish is characterized by a gastrulation arrest phenotype with cytoplasmic accumulation of E-cadherin.

Given that the knockdown of PrP1 or its molecular cousin ZIP6 gives rise to similar gastrulation defects in zebrafish, the question arises as to whether a primary role of the prion protein is to modulate ZIP-dependent cation import. The evolutionary relationship of PrP and ZIPs and the ability of ZIPs to interact with PrP^C^ are consistent with such a model (Schmitt-Ulms et al., 2009). However, close scrutiny of the precise E-cadherin deficiencies in the respective knockdown phenotypes may suggest a more complex scenario. Whereas the zinc import function of the aforementioned ZIPs appears to reduce E-cadherin levels at the transcriptional level (Yamashita et al., 2004), PrP deficiency in zebrafish does not seem to interfere with E-cadherin transcription but may prevent E-cadherin from reaching the plasma membrane (Malaga-Trillo et al., 2009; Solis et al., 2013). Thus, PrP/ZIPs may influence functional E-cadherin levels by a coordinated transcriptional and post-translational regulation. A plausible scenario (Figure 2) sees the zinc import function of ZIPs regulate E-cadherin gene transcription, possibly by controlling the zinc-dependent nuclear translocation of certain transcription factors such as the zinc finger EMT master regulator Snail (Yamashita et al., 2004). This signaling pathway may involve glycogen synthase kinase 3 beta (GSK-3β) (Hogstrand et al., 2013). PrP may also act as a modulator of this ZIP-dependent zinc import by acting as a scavenger or sensor of zinc ions (Watt et al., 2013), or on account of its ability to acquire alternative N-terminal folds in response to changes in its surrounding cation milieu (Chattopadhyay et al., 2005). Metal-dependent conformational changes of PrP could, for instance, modulate access of divalent cations to the ion channel present in a nearby ZIP protein, either by sterically blocking the channel or by exerting an influence on the arrangement of the channel transmembrane domains. On the other hand, PrP may serve a role in controlling functional E-cadherin levels post-translationally through the activation of Fyn (Lilien and Balsamo, 2005; Smyth et al., 2012). Assuming that PrP inherited this property from its ZIP ancestor, and that it can still be found in contemporary ZIP transporters equipped with a PL ectodomain, these ZIP molecules may operate as highly specialized regulators of EMT. It will be interesting to explore if the PL ectodomain of ZIPs can influence Fyn activation similar to PrP, and if such dual capability is at play in EMT paradigms that lack the prion protein. An example could be the process of gonad formation in *Drosophila*, where the ZIP paralog FOI is known to play a critical role in modulating E-cadherin expression and stability (Jenkins et al., 2003; Van Doren et al., 2003; Mathews et al., 2006). Indeed, a first indication that FOI might influence E-cadherin biology at the transcriptional and post-translational levels emerged from elegant *in vivo* functional rescue experiments conducted with FOI-knockout flies. In contrast to the aforementioned ZIP6 zebrafish gastrulation paradigm, mutant zinc import-defective derivatives of FOI were, observed to exhibit reduced DE-cadherin expression. Significantly, forced expression of DE-cadherin from an unrelated tubulin promoter still did not lead to a functional rescue unless FOI was also present to ensure the post-translational stabilization of DE-cadherin (Mathews et al., 2006).

## Conclusions

During the course of EMT, cells have to master the feat of gradually shifting their cell-cell and cell-substrate contacts from a stably adherent to a more transient and focal mode of attachment. The cellular program underlying these changes requires the concerted action of many molecules. Here we reviewed evidence consistent with the notion that members of the ZIP/PrP protein family may influence this program by affecting E-cadherin biology at multiple levels. It is likely that the cellular context will play a critical role for the direction and extent to which the expression of ZIPs/PrP affects this biology. With the advent of novel genome editing technologies and advances in quantitative protein mass spectrometry, the involvement of PrP in EMT and related cellular processes can now be studied much more elegantly. It is to be expected that these efforts will in the not-too-distant future lead to a much more detailed understanding of the cellular pathways that underlie phenotypic changes observed in PrP knockout paradigms and, ultimately, reveal the molecular function of this protein.

### Conflict of interest statement

The authors declare that the research was conducted in the absence of any commercial or financial relationships that could be construed as a potential conflict of interest.

## Abbreviations

CRISPR: clustered regularly interspaced short palindromic repeats
EMT: epithelial-to-mesenchymal transition
FOI: Fear-Of-Intimacy
GSK-3β: glycogen synthase kinase 3 beta
MAP: mitogen-activated protein
NCAM: neural cell adhesion molecule
PL: PrP-like
PrP^C^: cellular prion protein
SFK: Src family kinase
TGFβ: transforming growth factor beta
ZIP: Zrt-, Irt-like Protein.

## References

[B1] Aguzzi A. Baumann F. Bremer J. (2008). The Prion's elusive reason for being. Annu. Rev. Neurosci. 31, 439–477. 10.1146/annurev.neuro.31.060407.125620 18558863

[B2] Antonacopoulou A. G. Palli M. Marousi S. Dimitrakopoulos F. I. Kyriakopoulou U. Tsamandas A. C.. (2010). Prion protein expression and the M129V polymorphism of the PRNP gene in patients with colorectal cancer. Mol. Carcinog. 49, 693–699. 10.1002/mc.20642 20564346

[B3] Bremer J. Baumann F. Tiberi C. Wessig C. Fischer H. Schwarz P.. (2010). Axonal prion protein is required for peripheral myelin maintenance. Nat. Neurosci. 13, 310–318. 10.1038/nn.2483 20098419

[B4] Chattopadhyay M. Walter E. D. Newell D. J. Jackson P. J. Aronoff-Spencer E. Peisach J.. (2005). The octarepeat domain of the prion protein binds Cu(II) with three distinct coordination modes at pH 7.4. J. Am. Chem. Soc. 127, 12647–12656. 10.1021/ja053254z 16144413 PMC2909831

[B5] Colling S. B. Khana M. Collinge J. Jefferys J. G. (1997). Mossy fibre reorganization in the hippocampus of prion protein null mice. Brain Res. 755, 28–35. 10.1016/S0006-8993(97)00087-5 9163538

[B6] Crusio W. E. (2002). My mouse has no phenotype. Genes Brain Behav. 1, 71. 10.1034/j.1601-183X.2002.10201.x 12884976

[B7] Du L. Rao G. Wang H. Li B. Tian W. Cui J. T.. (2013). CD44-positive cancer stem cells expressing cellular prion protein contribute to metastatic capacity in colorectal cancer. Cancer Res. 73, 2682–2694. 10.1158/0008-5472.CAN-12-3759 23418321

[B8] Ehsani S. Mehrabian M. Pocanschi C. L. Schmitt-Ulms G. (2012). The ZIP-prion connection. Prion 6, 317–321. 10.4161/pri.20196 22575750 PMC3609057

[B9] Ehsani S. Tao R. Pocanschi C. L. Ren H. Harrison P. M. Schmitt-Ulms G. (2011). Evidence for retrogene origins of the prion gene family. PLoS ONE 6:e26800. 10.1371/journal.pone.0026800 22046361 PMC3203146

[B10] Evseenko D. Zhu Y. Schenke-Layland K. Kuo J. Latour B. Ge S.. (2010). Mapping the first stages of mesoderm commitment during differentiation of human embryonic stem cells. Proc. Natl. Acad. Sci. U.S.A. 107, 13742–13747. 10.1073/pnas.1002077107 20643952 PMC2922221

[B11] Gauczynski S. Peyrin J. M. Haik S. Leucht C. Hundt C. Rieger R.. (2001). The 37-kDa/67-kDa laminin receptor acts as the cell-surface receptor for the cellular prion protein. EMBO J. 20, 5863–5875. 10.1093/emboj/20.21.5863 11689427 PMC125290

[B12] Godt D. Tepass U. (2003). Organogenesis: keeping in touch with the germ cells. Curr. Biol. 13, R683–R685. 10.1016/S0960-9822(03)00609-2 12956974

[B13] Guo H. Jin X. Zhu T. Wang T. Tong P. Tian L.. (2014). SLC39A5 mutations interfering with the BMP/TGF-beta pathway in non-syndromic high myopia. J. Med. Genet. 51, 518–525. 10.1136/jmedgenet-2014-102351 24891338 PMC4112430

[B14] Hogstrand C. Kille P. Ackland M. L. Hiscox S. Taylor K. M. (2013). A mechanism for epithelial-mesenchymal transition and anoikis resistance in breast cancer triggered by Zinc channel zip6 and signal transducer and activator of transcription 3 (STAT3). Biochem. J. 455, 229–237. 10.1042/bj20130483 23919497 PMC3789231

[B15] Hsu P. D. Lander E. S. Zhang F. (2014). Development and applications of CRISPR-Cas9 for genome engineering. Cell 157, 1262–1278. 10.1016/j.cell.2014.05.010 24906146 PMC4343198

[B16] Jenkins A. B. McCaffery J. M. Van Doren M. (2003). Drosophila E-cadherin is essential for proper germ cell-soma interaction during gonad morphogenesis. Development 130, 4417–4426. 10.1242/dev.00639 12900457

[B17] Kiefel H. Bondong S. Pfeifer M. Schirmer U. Erbe-Hoffmann N. Schafer H.. (2012). EMT-associated up-regulation of L1CAM provides insights into L1CAM-mediated integrin signalling and NF-kappaB activation. Carcinogenesis 33, 1919–1929. 10.1093/carcin/bgs220 22764136

[B18] Lamouille S. Xu J. Derynck R. (2014). Molecular mechanisms of epithelial-mesenchymal transition. Nat. Rev. Mol. Cell Biol. 15, 178–196. 10.1038/nrm3758 24556840 PMC4240281

[B19] Lehembre F. Yilmaz M. Wicki A. Schomber T. Strittmatter K. Ziegler D.. (2008). NCAM-induced focal adhesion assembly: a functional switch upon loss of E-cadherin. EMBO J. 27, 2603–2615. 10.1038/emboj.2008.178 18772882 PMC2567408

[B20] Li J. J. Xie D. (2014). RACK1, a versatile hub in cancer. Oncogene. [Epub ahead of print]. 10.1038/onc.2014.127 24882575

[B21] Lichten L. A. Cousins R. J. (2009). Mammalian zinc transporters: nutritional and physiologic regulation. Annu. Rev. Nutr. 29, 153–176. 10.1146/annurev-nutr-033009-083312 19400752

[B22] Lilien J. Balsamo J. (2005). The regulation of cadherin-mediated adhesion by tyrosine phosphorylation/dephosphorylation of beta-catenin. Curr. Opin. Cell Biol. 17, 459–465. 10.1016/j.ceb.2005.08.009 16099633

[B23] Lim J. Thiery J. P. (2012). Epithelial-mesenchymal transitions: insights from development. Development 139, 3471–3486. 10.1242/dev.071209 22949611

[B24] Lin Z. Fang D. (2013). The Roles of SIRT1 in Cancer. Genes Cancer 4, 97–104. 10.1177/1947601912475079 24020000 PMC3764469

[B25] Malaga-Trillo E. Sempou E. (2009). PrPs: proteins with a purpose: lessons from the zebrafish. Prion 3, 129–133. 10.4161/pri.3.3.9651 19786844 PMC2802776

[B26] Malaga-Trillo E. Solis G. P. Schrock Y. Geiss C. Luncz L. Thomanetz V.. (2009). Regulation of embryonic cell adhesion by the prion protein. PLoS Biol. 7:e55. 10.1371/journal.pbio.1000055 19278297 PMC2653553

[B27] Mathews W. R. Ong D. Milutinovich A. B. Van Doren M. (2006). Zinc transport activity of fear of Intimacy is essential for proper gonad morphogenesis and DE-cadherin expression. Development 133, 1143–1153. 10.1242/dev.02256 16481356

[B28] Mathews W. R. Wang F. Eide D. J. Van Doren M. (2005). Drosophila fear of intimacy encodes a Zrt/IRT-like protein (ZIP) family zinc transporter functionally related to mammalian ZIP proteins. J. Biol. Chem. 280, 787–795. 10.1074/jbc.M411308200 15509557

[B29] McDonald S. L. Silver A. (2009). The opposing roles of Wnt-5a in cancer. Br. J. Cancer 101, 209–214. 10.1038/sj.bjc.6605174 19603030 PMC2720208

[B30] Mehrpour M. Codogno P. (2010). Prion protein: from physiology to cancer biology. Cancer Lett. 290, 1–23. 10.1016/j.canlet.2009.07.009 19674833

[B31] Mouillet-Richard S. Ermonval M. Chebassier C. Laplanche J. L. Lehmann S. Launay J. M.. (2000). Signal transduction through prion protein. Science 289, 1925–1928. 10.1126/science.289.5486.1925 10988071

[B32] Mu D. Cambier S. Fjellbirkeland L. Baron J. L. Munger J. S. Kawakatsu H.. (2002). The integrin alpha(v)beta8 mediates epithelial homeostasis through MT1-MMP-dependent activation of TGF-beta1. J. Cell Biol. 157, 493–507. 10.1083/jcb.200109100 11970960 PMC2173277

[B33] Munger J. S. Huang X. Kawakatsu H. Griffiths M. J. Dalton S. L. Wu J.. (1999). The integrin alpha v beta 6 binds and activates latent TGF beta 1: a mechanism for regulating pulmonary inflammation and fibrosis. Cell 96, 319–328. 10.1016/S0092-8674(00)80545-0 10025398

[B34] Naslavsky N. Stein R. Yanai A. Friedlander G. Taraboulos A. (1997). Characterization of detergent-insoluble complexes containing the cellular prion protein and its scrapie isoform. J. Biol. Chem. 272, 6324–6331. 10.1074/jbc.272.10.6324 9045652

[B35] Niethammer P. Delling M. Sytnyk V. Dityatev A. Fukami K. Schachner M. (2002). Cosignaling of NCAM via lipid rafts and the FGF receptor is required for neuritogenesis. J. Cell Biol. 157, 521–532. 10.1083/jcb.200109059 11980923 PMC2173281

[B36] Pan Y. Zhao L. Liang J. Liu J. Shi Y. Liu N.. (2006). Cellular prion protein promotes invasion and metastasis of gastric cancer. FASEB J. 20, 1886–1888. 10.1096/fj.06-6138fje 16877520

[B37] Pantera B. Bini C. Cirri P. Paoli P. Camici G. Manao G.. (2009). PrPc activation induces neurite outgrowth and differentiation in PC12 cells: role for caveolin-1 in the signal transduction pathway. J. Neurochem. 110, 194–207. 10.1111/j.1471-4159.2009.06123.x 19457127

[B38] Rubenstein R. (2012). Proteomic analysis of prion diseases: creating clarity or causing confusion? Electrophoresis 33, 3631–3643. 10.1002/elps.201200310 23161058

[B39] Sailer A. Bueler H. Fischer M. Aguzzi A. Weissmann C. (1994). No propagation of prions in mice devoid of PrP. Cell 77, 967–968. 10.1016/0092-8674(94)90436-7 7912659

[B40] Santuccione A. Sytnyk V. Leshchyns'ka I. Schachner M. (2005). Prion protein recruits its neuronal receptor NCAM to lipid rafts to activate p59fyn and to enhance neurite outgrowth. J. Cell Biol. 169, 341–354. 10.1083/jcb.200409127 15851519 PMC2171870

[B41] Schmitt-Ulms G. Ehsani S. Watts J. C. Westaway D. Wille H. (2009). Evolutionary descent of prion genes from the ZIP family of metal ion transporters. PLoS ONE 4:e7208. 10.1371/journal.pone.0007208 19784368 PMC2745754

[B42] Shen H. Qin H. Guo J. (2009). Concordant correlation of LIV-1 and E-cadherin expression in human breast cancer cell MCF-7. Mol. Biol. Rep. 36, 653–659. 10.1007/s11033-008-9225-4 18330719

[B43] Smyth D. Leung G. Fernando M. McKay D. M. (2012). Reduced surface expression of epithelial E-cadherin evoked by interferon-gamma is Fyn kinase-dependent. PLoS ONE 7:e38441. 10.1371/journal.pone.0038441 22715382 PMC3371038

[B44] Solis G. P. Radon Y. Sempou E. Jechow K. Stuermer C. A. Malaga-Trillo E. (2013). Conserved roles of the prion protein domains on subcellular localization and cell-cell adhesion. PLoS ONE 8:e70327. 10.1371/journal.pone.0070327 23936187 PMC3729945

[B45] Steele A. D. Lindquist S. Aguzzi A. (2007). The prion protein knockout mouse: a phenotype under challenge. Prion 1, 83–93. 10.4161/pri.1.2.4346 19164918 PMC2634447

[B46] Thiery J. P. Acloque H. Huang R. Y. Nieto M. A. (2009). Epithelial-mesenchymal transitions in development and disease. Cell 139, 871–890. 10.1016/j.cell.2009.11.007 19945376

[B47] Tobler I. Gaus S. E. Deboer T. Achermann P. Fischer M. Rulicke T.. (1996). Altered circadian activity rhythms and sleep in mice devoid of prion protein. Nature 380, 639–642. 10.1038/380639a0 8602267

[B48] Tomasi V. (2010). Signal transduction in neurons: effects of cellular prion protein on fyn kinase and ERK1/2 kinase. Immun. Ageing 7, S5. 10.1186/1742-4933-7-S1-S5 21172064 PMC3024879

[B49] Toni M. Spisni E. Griffoni C. Santi S. Riccio M. Lenaz P.. (2006). Cellular prion protein and caveolin-1 interaction in a neuronal cell line precedes Fyn/Erk 1/2 signal transduction. J. Biomed. Biotechnol. 2006, 1–13. 10.1155/JBB/2006/69469 17489019 PMC1559926

[B50] Um J. W. Nygaard H. B. Heiss J. K. Kostylev M. A. Stagi M. Vortmeyer A.. (2012). Alzheimer amyloid-beta oligomer bound to postsynaptic prion protein activates Fyn to impair neurons. Nat. Neurosci. 15, 1227–1235. 10.1038/nn.3178 22820466 PMC3431439

[B51] Van Doren M. Mathews W. R. Samuels M. Moore L. A. Broihier H. T. Lehmann R. (2003). Fear of intimacy encodes a novel transmembrane protein required for gonad morphogenesis in Drosophila. Development 130, 2355–2364. 10.1242/dev.00454 12702650

[B52] Walsh F. S. Parekh R. B. Moore S. E. Dickson G. Barton C. H. Gower H. J.. (1989). Tissue specific O-linked glycosylation of the neural cell adhesion molecule (N-CAM). Development 105, 803–811. 2598815 10.1242/dev.105.4.803

[B53] Wang Z. Liu P. Inuzuka H. Wei W. (2014). Roles of F-box proteins in cancer. Nat. Rev. Cancer 14, 233–247. 10.1038/nrc3700 24658274 PMC4306233

[B54] Watt N. T. Griffiths H. H. Hooper N. M. (2013). Neuronal zinc regulation and the prion protein. Prion 7, 203–208. 10.4161/pri.24503 23764834 PMC3783104

[B55] Watts J. C. Huo H. Bai Y. Ehsani S. Jeon A. H. Shi T.. (2009). Interactome analyses identify ties of PrP and its mammalian paralogs to oligomannosidic N-glycans and endoplasmic reticulum-derived chaperones. PLoS Pathog. 5:e1000608. 10.1371/journal.ppat.1000608 19798432 PMC2749441

[B56] Watts J. C. Westaway D. (2007). The prion protein family: diversity, rivalry, and dysfunction. Biochim. Biophys. Acta 1772, 654–672. 10.1016/j.bbadis.2007.05.001 17562432

[B57] Wurm S. Wechselberger C. (2006). Prion protein modifies TGF-beta induced signal transduction. Biochem. Biophys. Res. Commun. 349, 525–532. 10.1016/j.bbrc.2006.08.074 16942751

[B58] Yamashita S. Miyagi C. Fukada T. Kagara N. Che Y. S. Hirano T. (2004). Zinc transporter LIVI controls epithelial-mesenchymal transition in zebrafish gastrula organizer. Nature 429, 298–302. 10.1038/nature02545 15129296

[B59] Yang Z. Mu Z. Dabovic B. Jurukovski V. Yu D. Sung J.. (2007). Absence of integrin-mediated TGFbeta1 activation *in vivo* recapitulates the phenotype of TGFbeta1-null mice. J. Cell Biol. 176, 787–793. 10.1083/jcb.200611044 17353357 PMC2064053

[B60] Zhang C. C. Steele A. D. Lindquist S. Lodish H. F. (2006). Prion protein is expressed on long-term repopulating hematopoietic stem cells and is important for their self-renewal. Proc. Natl. Acad. Sci. U.S.A. 103, 2184–2189. 10.1073/pnas.0510577103 16467153 PMC1413720

[B61] Zomosa-Signoret V. Arnaud J. D. Fontes P. Alvarez-Martinez M. T. Liautard J. P. (2008). Physiological role of the cellular prion protein. Vet. Res. 39, 9. 10.1051/vetres:2007048 18073096

